# Microsurgical Untethering of Pediatric Lipomyelomeningocele: A Stepwise, Photo-Illustrated Technical Note

**DOI:** 10.3390/brainsci16070720

**Published:** 2026-07-05

**Authors:** Chul Ou Lee, Kwan-Sung Lee, Seung Ho Yang

**Affiliations:** Department of Neurosurgery, Seoul St. Mary’s Hospital, College of Medicine, The Catholic University of Korea, 222 Banpodaero, Seocho-gu, Seoul 06591, Republic of Korea

**Keywords:** spinal dysraphism, lipoma, neurosurgical procedures, child, microsurgery

## Abstract

**Highlights:**

**What are the main findings?**

- We describe a stepwise, 14-step microsurgical technique for pediatric lipomyelomeningocele (LMMC), anchored to 15 sequential intra-operative photographs and explicit anatomical landmarks—the dural penetration site, the arachnoid–dura plane, the lipoma–placode interface, and the lipoma-laden filum terminale—that operationalizes radical untethering with pia-to-pia placode reconstruction and expansile duraplasty.
- Each step incorporates a defined technical pearl: incision planning away from the anal verge, opening the dura over normal anatomy first, electrophysiologically guided lipoma debulking, stimulation-controlled division of the fatty filum, and reinforced multilayered watertight closure with an artificial dural substitute and a sealant patch.

**What are the implications of the main findings?**

- By translating contemporary radical-untethering principles into a concrete, image-anchored 14-step roadmap, the technique simultaneously targets the three principal modes of post-operative failure—direct neural injury, cerebrospinal fluid leak, and symptomatic re-tethering—and provides a practical operative reference for pediatric neurosurgeons and trainees managing this challenging closed neural tube defect.
- Routine integration of multimodal intraoperative neurophysiological monitoring with direct stimulation before any division of presumed lipomatous tissue allows aggressive but safe debulking, supporting the wider adoption of placode-preserving radical resection over historical partial resection, which has been associated with re-tethering rates of 15–25%.

**Abstract:**

Lipomyelomeningocele (LMMC) is one of the most common forms of occult spinal dysraphism, with an estimated incidence of 3–6 per 100,000 live births, and microsurgical untethering remains the cornerstone of management for symptomatic and selected at-risk children. The operation is technically demanding: reported rates of long-term symptomatic re-tethering after partial resection still reach 15–25%, and the surgeon must balance adequate untethering against preservation of the placode and lumbosacral nerve roots. In this technical note, we present a stepwise, illustrated description of our institutional 14-step microsurgical technique for pediatric LMMC. Each step is anchored to a defined anatomical landmark, beginning with a midline skin incision planned away from the anal verge and proceeding through subtotal subcutaneous lipoma resection, identification of the dural penetration site, a limited rostral laminectomy over normal anatomy, dural opening with circumferential dissection of lipoma–dura–cord adhesions, exploitation of the arachnoid–dura plane, electrophysiologically guided debulking of the intradural lipoma, stimulation-controlled division of the fatty filum, pia-to-pia reconstruction of the placode with 8-0 monofilament suture, expansile duraplasty with an artificial dural substitute, and reinforced multilayered watertight closure. Technical pearls aimed at minimizing the risks of cord injury, cerebrospinal fluid leak, and postoperative re-tethering are highlighted at each stage, and the role of multimodal intraoperative neurophysiological monitoring is emphasized. This note is intended as a practical, image-anchored operative reference for pediatric neurosurgeons and trainees managing this challenging closed neural tube defect.

## 1. Introduction

Lipomyelomeningocele (LMMC) is a closed neural tube defect in which a subcutaneous lipoma extends through a fascial and dural defect to attach to a low-lying, tethered spinal cord at the neural placode [1,2]. It is one of the most common forms of occult spinal dysraphism, with an estimated incidence of 3–6 per 100,000 live births [3]. The pathogenesis involves a failure of primary neurulation, with proposed sub-classifications according to the lipoma–cord interface and to the embryological stage of the defect, which together inform surgical complexity and prognosis [3,4,5]. Recently, the international Spina Bifida and other Dysraphisms (SBoD) working group has issued a Delphi-derived revision of the Orphanet nomenclature, in which descriptive anatomical findings replace embryological labels; what is described here as LMMC most often corresponds to the posterior extramedullary conus spinal cord lipoma (ORPHA:645294) or the terminal extramedullary conus spinal cord lipoma (ORPHA:645288) in that framework [6]. We retain the term “lipomyelomeningocele” for continuity with the surgical literature, while explicitly mapping it to this updated classification. Although there is ongoing debate regarding the role of prophylactic surgery in fully asymptomatic patients, contemporary meta-analyses and large series support surgical untethering for symptomatic children and at-risk infants, with the goals of preventing or arresting progressive neurological, urological, and orthopedic deterioration [7,8,9,10]. Reported rates of symptomatic re-tethering after partial resection are approximately 15–25% and have driven the evolution of more radical resection and meticulous placode reconstruction techniques [1,11,12,13].

This technical note describes our institutional step-by-step technique for the resection and untethering of pediatric LMMC, with emphasis on the surgical landmarks identified at each stage and on the technical maneuvers used to minimize the risks of cord injury, cerebrospinal fluid (CSF) leak, and re-tethering [11,14,15].

## 2. Preoperative Preparation and Positioning

Cutaneous stigmata such as a lumbosacral subcutaneous mass, dimple, hairy patch, or capillary hemangioma should prompt early imaging; the prevalence of occult spinal dysraphism in infants with cutaneous stigmata is meaningful and warrants careful screening [16].

**Figure 1:**
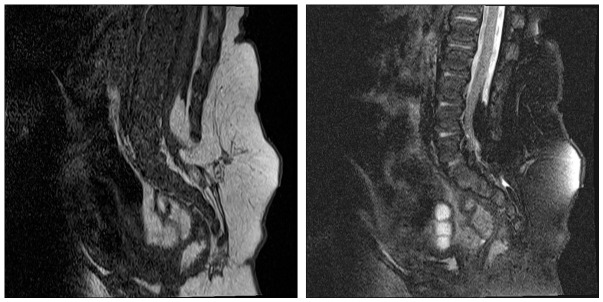
Pre-operative sagittal magnetic resonance imaging of the lumbosacral spine. **Left**: T1-weighted image, demonstrating the high-signal subcutaneous lipoma extending posteriorly from the spinal canal through a fascial defect; the lipoma is in continuity with intradural fat that attaches to a low-lying, tethered neural placode. **Right**: T2-weighted image of the same patient, demonstrating the conus medullaris position, the cord–sac relationship, and the cerebrospinal fluid-containing subarachnoid space.

Preoperative magnetic resonance imaging (MRI) of the entire spine is mandatory in every case and defines the level and morphology of the lipoma, the position of the conus, the cord–sac ratio, the side of the placode–lipoma interface, and the relationship of the nerve roots; an associated syrinx should be specifically sought, as its presence has been associated with worse preoperative function in LMMC (Figure 1) [10,17].

Baseline neurological and urodynamic evaluations are obtained whenever feasible, since urological function is a sensitive marker of long-term outcome after untethering [9,14].

General endotracheal anesthesia is induced. Long-acting muscle relaxants are avoided after induction to preserve reliable intraoperative neurophysiological monitoring (IONM), including motor evoked potentials (MEPs), free-running EMG, and the bulbocavernosus reflex [12,13,18].

The patient is placed prone with chest and pelvic rolls and the abdomen freely hanging to reduce epidural venous engorgement. The buttocks and perineum are sealed off from the operative field with an adhesive drape, and a Foley catheter is placed.

A direct-current handheld nerve stimulator, operating microscope, and microsurgical instruments are prepared. An 8-0 monofilament non-absorbable suture (e.g., Ethilon^®^ 8-0; Ethicon, Somerville, NJ, USA) and an autologous duraplasty substrate (lumbosacral fascia or pericranium), with an acellular biological dural xenograft (e.g., Surgisis^®^, Cook Medical Inc., Bloomington, IN, USA) as a back-up, are made available before draping [11,15].

## 3. Surgical Procedure

### 3.1. Step 1. Skin Incision and Exposure of the Subcutaneous Lipoma

**Figure 2:**
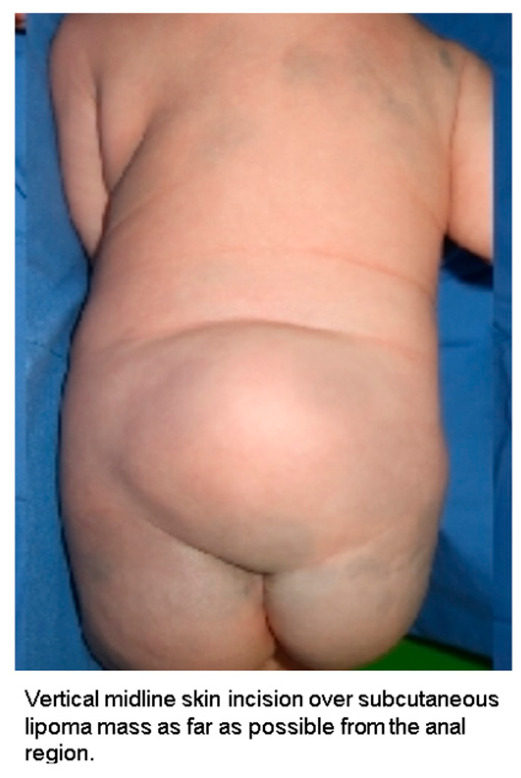
Pre-operative external appearance of a pediatric patient with lipomyelomeningocele, demonstrating the lumbosacral subcutaneous lipomatous mass and the planned vertical midline skin incision extending along the long axis of the lipoma, ending as far as possible from the anal verge.

A vertical fusiform elliptical skin incision is centered on the subcutaneous lipoma. The incision is planned to extend along the long axis of the lipoma, beginning above the rostral pole of the mass and ending as far as possible from the anal verge to reduce the risk of wound contamination and dehiscence (Figure 2) [11]. A transverse skin incision may be considered as an alternative in low-lying lesions that lie close to the gluteal cleft, since a transverse scar may be less prone to contamination in the diaper area; in our experience, however, a vertical incision provides better exposure of the rostral pole of the lipoma where the dural penetration site is identified. Skin and subcutaneous fat are sharply incised, and the lobulated, pale-yellow lipoma is identified in continuity with normal subcutaneous fat.

### 3.2. Step 2. Subtotal Resection of the Subcutaneous Lipoma and Identification of the Fascial Defect

**Figure 3:**
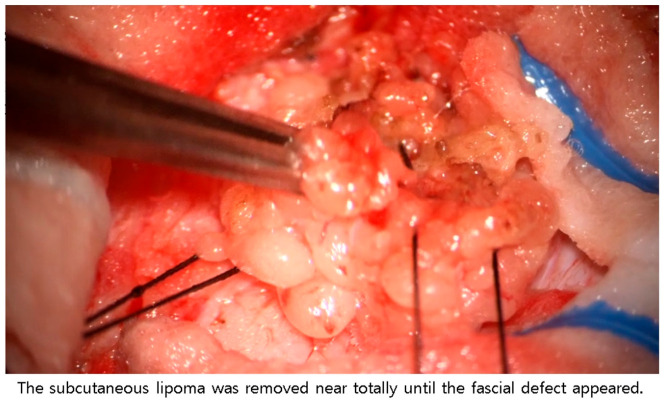
The subcutaneous lipoma is removed near-totally until the fascial defect appears.

The bulk of the subcutaneous lipoma is debulked using bipolar cautery and sharp dissection. Resection is carried down circumferentially until the normal lumbosacral fascia is encountered on all sides and the fascial defect through which the lipoma exits is clearly delineated (Figure 3). Aggressive subcutaneous resection beyond what is needed to expose the fascial defect is avoided, both to limit dead space (and thus the risk of pseudomeningocele) and to preserve a vascularized soft-tissue envelope for closure [11,15].

### 3.3. Step 3. Identification of the Dural Penetration of the Lipoma

**Figure 4:**
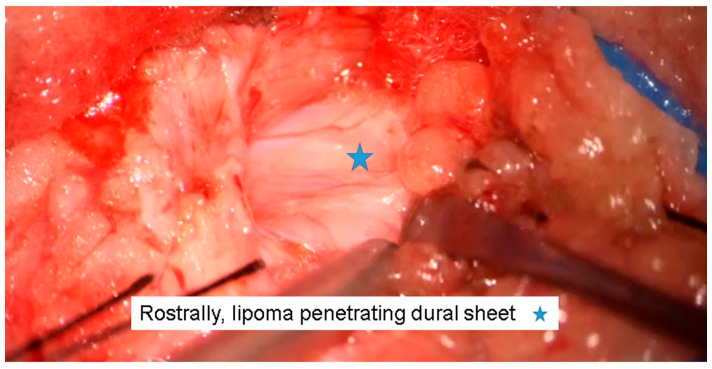
Rostrally, the lipoma is seen penetrating the dural sheet (★).

Once the fascial defect is exposed with rostral extension of at least one vertebral segment beyond the defect, the lipoma is followed proximally (rostrally) until its dural penetration site is identified (Figure 4). This point—the transition from the extradural to the intradural component—is the key landmark for planning the laminectomy.

### 3.4. Step 4. Laminectomy to Expose the Rostral End of the Intradural Lipoma

**Figure 5:**
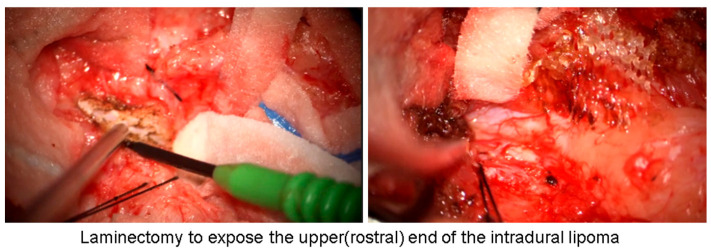
Laminectomy performed to expose the upper (rostral) end of the intradural lipoma.

A bilateral laminectomy is performed at the level immediately rostral to the dural defect, until normal dura is identified (Figure 5). This single-level laminectomy is necessary to expose a segment of intact, healthy dura over normal-caliber spinal cord through which the dural opening can begin safely; because the lumbar laminae at the level of the dysraphism are already congenitally deficient (osseous schisis), the additional minimal exposure one level above the defect represents a small incremental bony disruption that, in our experience, has not been associated with clinically significant post-laminectomy spinal deformity in this population. The exposure is carried just far enough cephalad to identify a segment of intact, healthy dura covering normal-caliber spinal cord above the lipoma. Beginning the work over normal anatomy is essential because it allows the surgeon to enter the dura safely and proceed from known territory into the abnormal segment [11].

### 3.5. Step 5. Rostral Dural Opening and Dissection of Lipoma–Dura–Cord Adhesions

**Figure 6:**
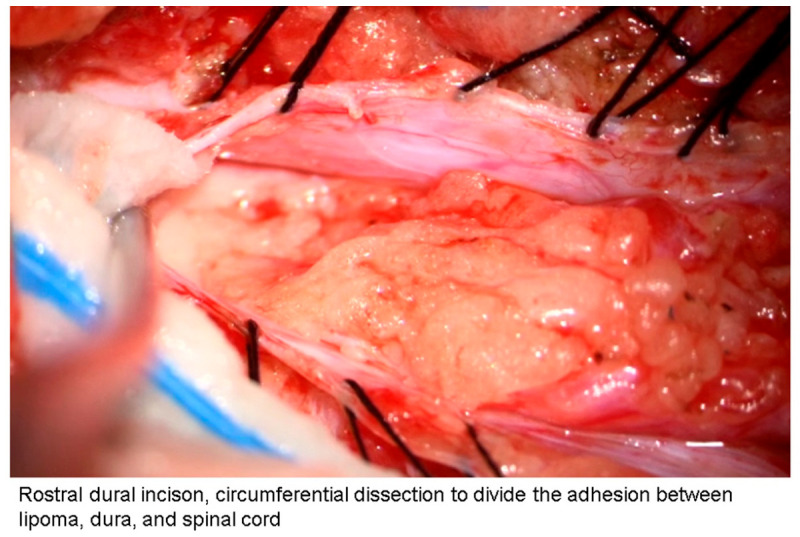
Rostral dural incision and circumferential dissection to divide the adhesions between lipoma, dura, and spinal cord.

The dura is opened in the midline beginning rostrally over normal cord and extended caudally toward the lipoma. The dural edges are tacked up with sutures. Under microscopic magnification, the rostral pole of the intradural lipoma is dissected free circumferentially from its adhesions to the dura and to the dorsal surface of the spinal cord using sharp microscissor dissection supplemented by fine bipolar coagulation on low settings (Figure 6). This stepwise approach is consistent with the principles described for radical lipoma resection and placode reconstruction [11,15].

### 3.6. Step 6. Dissection in the Plane Between the Arachnoid and the Dura

**Figure 7:**
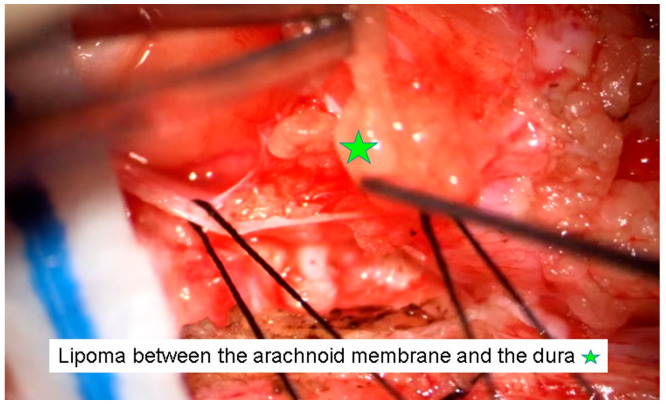
The lipoma (★) lies between the arachnoid membrane and the dura, providing a useful surgical plane.

An anatomical observation that facilitates safe dissection is that the lipoma frequently occupies the plane between the arachnoid and the inner surface of the dura, rather than within the subarachnoid space itself (Figure 7). Identifying and following this arachnoid–dura plane allows the lipoma to be peeled away from the dura while keeping underlying neural elements protected beneath the arachnoid [11].

### 3.7. Step 7. Caudal Extension of Dissection and Exposure of the Nerve Roots

**Figure 8:**
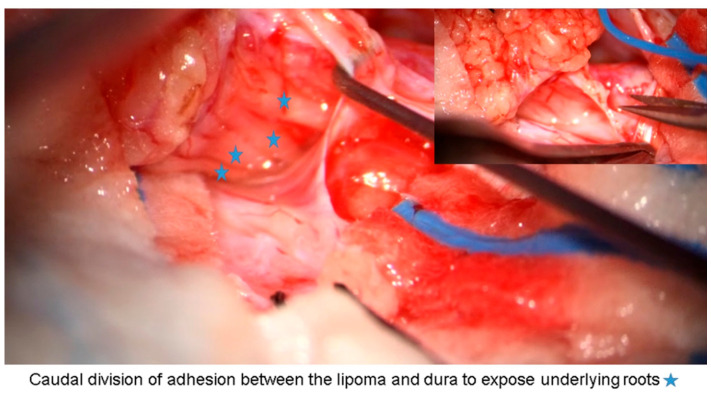
Caudal division of adhesion between the lipoma and dura exposes the underlying nerve roots (★).

Dissection is extended caudally along the lipoma–dura interface to free the lipoma from the dura on both sides. As the dissection advances, the nerve roots—particularly the sacral roots exiting toward the neural foramina—become visible beneath and lateral to the lipoma (Figure 8). Preservation of their anatomical integrity is paramount; intermittent direct stimulation with a handheld intraoperative nerve stimulator is performed to confirm the absence of functional neural elements within any tissue presumed to be lipoma prior to division. Multimodal IONM, comprising direct mapping and continuous MEP monitoring, has been demonstrated to be feasible and informative even in children younger than 2 years of age and to correlate with postoperative neurological outcome [12,13,18].

### 3.8. Step 8. Subtotal Resection of the Intradural Spinal Lipoma

**Figure 9:**
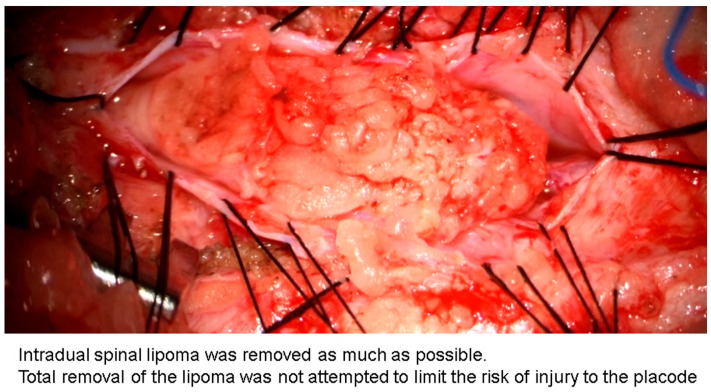
The intradural spinal lipoma is removed as much as safely possible. Total removal is not attempted, to limit the risk of injury to the placode.

The intradural component of the lipoma is then debulked (Figure 9). The extent of resection is tailored to the lipoma sub-type. In dorsal-type lipomas, where the cleavage plane between the placode and the lipoma is usually well defined, near-total or total resection is feasible and preferred. In transitional-type lipomas, in which the lipoma extends into the conus and the neural–fat interface is less distinct, debulking is carried out as far as the operator can safely dissect; total resection is not pursued at the cost of injuring functional neural tissue. In chaotic-type lipomas, where neural tissue is admixed with fat with no identifiable interface, only conservative internal debulking aimed at relieving traction is performed [3,11]. Resection is performed as aggressively as possible, but total removal is not pursued in our hands when adherence to the dorsal neural tissue is intimate, because the last few millimeters of fat at the cord interface confer a disproportionate risk of injury to functional neural tissue. The objective is to leave only a thin film of lipoma on the placode while debulking the bulk of the mass. Although total/near-total resection has been associated with lower long-term re-tethering and better progression-free survival in dedicated series [3,11], radical resection is technically demanding, and the optimal trade-off between extent of resection and functional preservation remains debated, particularly for transitional and chaotic lipomas [7,8,10].

### 3.9. Step 9. Identification of the Lipoma-Laden Filum and the Caudal End of the Placode

**Figure 10:**
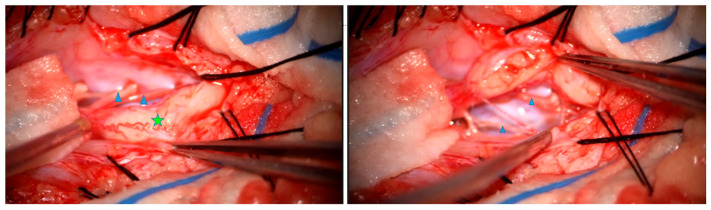
The lipoma in the filum (★) is observed at the caudal end of the placode; nerve roots exiting from the sacral neural foramina (▲) are identified.

After debulking, the caudal end of the placode is inspected (Figure 10). The fatty filum terminale, in continuity with the residual lipoma, is identified at the caudal pole. The sacral nerve roots exiting their respective neural foramina are again confirmed lateral to the placode and protected throughout the remainder of the procedure.

### 3.10. Step 10. Stimulation Testing and Division of the Filum Terminale

**Figure 11:**
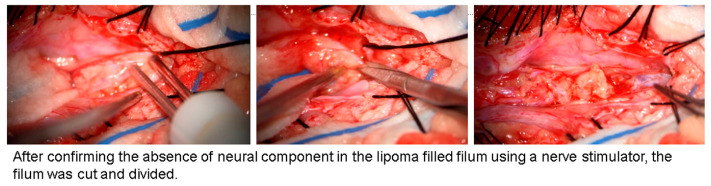
After confirming the absence of a neural component in the lipoma-filled filum using a nerve stimulator, the filum is divided.

Before division, the lipoma-laden filum is stimulated with the nerve stimulator at progressively higher currents to confirm the absence of any motor response, indicating that no functional neural component traverses it (Figure 11). Once silence is confirmed, the filum is coagulated and sharply divided, completing the untethering. Direct filum-terminale section is well established as a low-morbidity component of untethering surgery in pediatric patients [19].

### 3.11. Step 11. Pia-to-Pia Reconstruction of the Neural Placode

**Figure 12:**
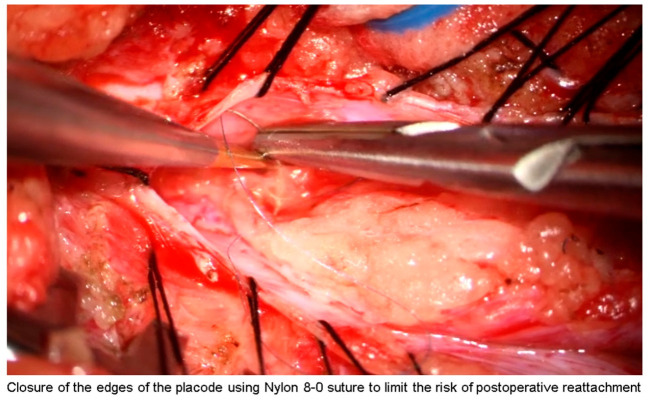
Closure of the edges of the placode using 8-0 nylon to limit the risk of postoperative re-tethering.

**Figure 13:**
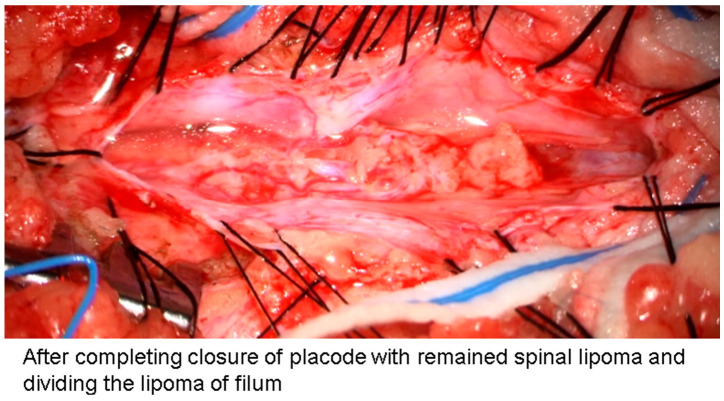
Final appearance after completion of placode closure with the residual spinal lipoma and division of the lipoma of the filum.

The lateral pial edges of the placode, now bearing only a thin film of residual lipoma, are reapproximated in the midline with interrupted 8-0 monofilament non-absorbable nylon sutures (e.g., Ethilon^®^ 8-0; Ethicon, Somerville, NJ, USA), recreating a tubular configuration (Figure 12). This pia-to-pia neurulation reduces the dorsal raw surface available for re-tethering, in keeping with the principles of meticulous placode reconstruction adopted in the literature [3,11,14,15]. Particular care is taken to avoid compression of the residual neural tissue and to ensure that no nerve roots are inadvertently incorporated within the suture line. Reactive non-monofilament materials such as silk are avoided. The final reconstructed placode is shown in Figure 13.

### 3.12. Step 12. Dural Closure with Augmentation Graft

**Figure 14:**
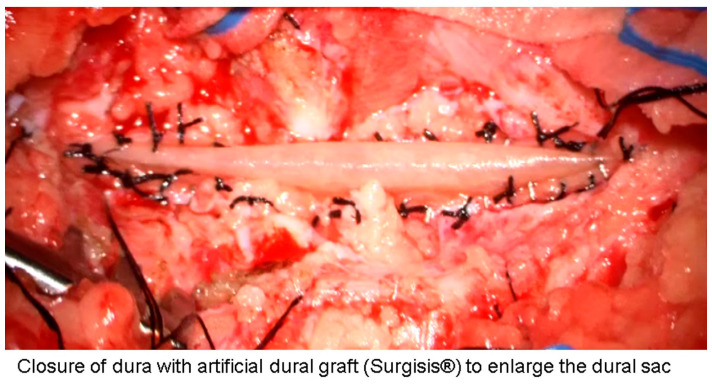
Closure of dura with autologous tissue or acellular biological xenograft to enlarge the dural sac (expansile duraplasty).

Because the native dura is often deficient and primary closure under tension would constrict the cord, the dura is closed in an expansile fashion to enlarge the dural sac and provide a tension-free, watertight closure (Figure 14). Autologous tissue (lumbosacral fascia or pericranium) is the preferred substrate for duraplasty in this setting and should be used whenever the size of the dural defect and the operative field permit. When sufficient autologous tissue cannot be harvested, an acellular biological xenograft (Surgisis^®^, Cook Medical Inc., Bloomington, IN, USA—porcine small intestinal submucosa, not a synthetic prosthesis) is used as a second-line substrate; truly synthetic prosthetic dural materials are avoided in this context. Expansile duraplasty has been advocated in radical lipoma surgery to create a capacious, low-cord–sac-ratio CSF reservoir thought to mitigate re-tethering [11,15].

### 3.13. Step 13. Reinforcement of the Dural Closure and Prevention of CSF Leak

**Figure 15:**
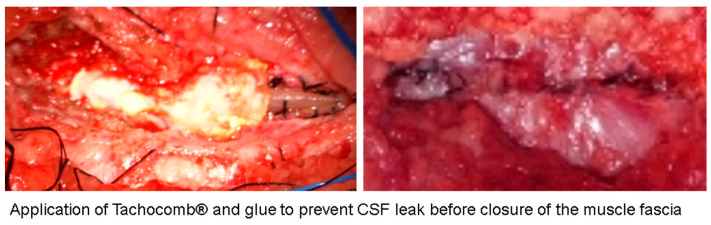
Application of TachoComb^®^ and fibrin glue to prevent CSF leak before closure of the muscle fascia.

A sealant patch (TachoComb^®^; CSL Behring K.K., Tokyo, Japan) is applied over the dural suture line, supplemented with fibrin sealant glue, to reinforce the closure and minimize the risk of postoperative CSF leak (Figure 15). A Valsalva maneuver is performed by the anesthesiologist to confirm watertight closure before proceeding to soft-tissue closure. Postoperative CSF leak and pseudomeningocele are recognized risk factors for re-tethering after complex spinal lipoma surgery [1,11].

### 3.14. Step 14. Soft-Tissue Closure

**Figure 16:**
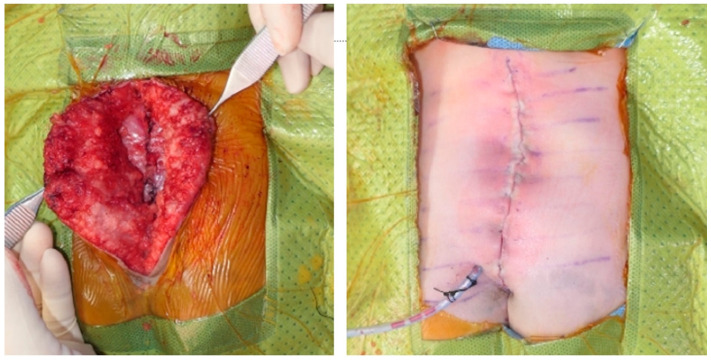
Layered fascia and skin closure. **Left**: deep layer; **Right**: completed midline vertical skin closure with drain.

The lumbosacral fascia is reapproximated meticulously in the midline with interrupted absorbable sutures to provide a strong second barrier against CSF leak. The subcutaneous tissue is closed in layers—over a closed-suction drain placed under direct vision, well away from the dural closure—and the skin is closed in a midline vertical fashion. Subcutaneous lipoma resection along a relatively long lumbosacral incision predictably creates a sizeable dead space, and large post-operative subcutaneous exudates and seromas have been reported in this setting [20], with one report describing more than 1000 mL of subcutaneous fluid requiring suction drainage. The drain is therefore used selectively to obliterate that dead space, to minimize sub-fascial fluid accumulation, and to reduce mechanical stress on the dural suture line. It is not positioned in continuity with the subarachnoid space and is removed once output declines (typically within 48–72 h) to limit any potential role as a route for retrograde contamination. A non-adherent dressing is applied, and the wound is kept away from the perineal region (Figure 16).

## 4. Postoperative Management

Patients are nursed in a slightly head-down prone position for approximately 3–5 days to reduce hydrostatic pressure on the dural closure.

Wound contamination from urine and stool is prevented with careful diapering, frequent changes, and an occlusive dressing where appropriate.

Neurological and urological status is documented preoperatively and reassessed serially after surgery; baseline urodynamic studies are obtained when feasible, as long-term urological outcome is the most sensitive measure of success after prophylactic untethering [9,14].

**Figure 17:**
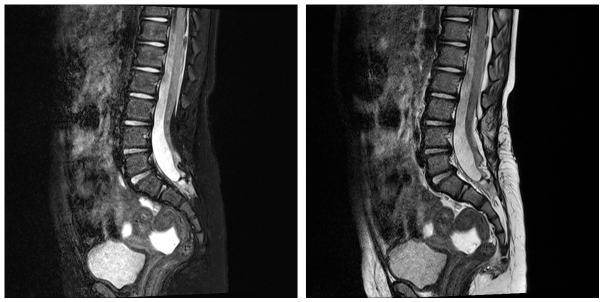
Post-operative sagittal MRI obtained one year after surgery. **Left**: T1-weighted image; **Right**: T2-weighted image. The intradural lipoma has been debulked, and the placode has been reconstructed and reduced to a tubular configuration; a capacious dural sac with a low cord–sac ratio is seen, consistent with an expansile duraplasty. No evidence of re-tethering, recurrent lipoma, or pseudomeningocele is identified.

Figure 17 shows follow-up MRI, which is typically obtained at 3 months and then periodically to monitor for re-tethering, residual lipoma growth, and pseudomeningocele [1].

## 5. Discussion

### 5.1. Key Technical Pearls

Plan the skin incision as far cephalad as the lesion permits and as far as possible from the anal verge to limit wound contamination [11].

Begin the dural opening over normal dura and normal cord rostral to the lipoma, and work down into the abnormal segment from above [11].

Recognize and exploit the plane between the lipoma and the dura—with the arachnoid often interposed between lipoma and cord—to minimize traction on neural elements.

Subtotal versus total resection should be tailored to the lipoma type and to operative findings: aggressive total resection improves long-term re-tethering rates in selected cases, but at the cost of greater technical difficulty, and is contraindicated when the lipoma–placode interface cannot be safely identified [3,4,5,11].

Confirm absence of functional neural elements by direct stimulation before dividing the filum terminale [12,13,19].

Use 8-0 monofilament non-absorbable suture for pia-to-pia placode reconstruction (neurulation) to reduce re-tethering [11,15].

Prefer expansile duraplasty over primary dural closure when there is dural insufficiency, to avoid iatrogenic cord constriction and create a capacious CSF reservoir [11,15].

Achieve layered watertight closure—dura with sealant, fascia, subcutaneous tissue, and skin—to prevent CSF leak and the cascade of pseudomeningocele, re-operation, and re-tethering [1,11,14].

### 5.2. Surgical Nuances by Lipoma Sub-Type

The 14-step framework described above is broadly applicable, but several specific decisions are sub-type dependent. In dorsal lipomas (Chapman type I; Pang “dorsal”), the lipoma–placode junction is typically a discrete, easily identifiable plane, and near-total intradural debulking with pia-to-pia closure is the standard goal; long-term re-tethering rates after radical resection are the lowest in this group [3,11]. In transitional lipomas, the lipoma extends into the substance of the conus and the cleavage plane is variable; here, we prioritise debulking until functional neural tissue is encountered intraoperatively (by direct stimulation), and we accept a thicker residual film of fat on the placode rather than risk a neurological deficit. In chaotic lipomas, in which lipoma and neural tissue are inseparably admixed, we limit ourselves to internal decompression and filum division for the purpose of relieving caudal traction; aggressive cleavage of a non-existent plane is contraindicated.

### 5.3. Updated Nomenclature and the Orphanet SBoD Classification

In 2025, the international SBoD working group published a Delphi-derived revision of the Orphanet nomenclature for spinal dysraphism, in which descriptive anatomical findings (skin, bone, spinal cord) replace embryological labels [6]. Most cases historically diagnosed as “lipomyelomeningocele” map to either the posterior extramedullary conus spinal cord lipoma (ORPHA:645294) or the terminal extramedullary conus spinal cord lipoma (ORPHA:645288). For this technical note we retain the conventional surgical term because it remains predominant in the operative literature, but we explicitly cross-reference the new framework to facilitate concordant indexing and future cross-study comparison. In the second round of peer review, Reviewer 3 reiterated the request to replace “lipomyelomeningocele” with “closed spinal dysraphism” throughout the manuscript. We have carefully considered this proposal but have respectfully retained the original term, with the following reasoning. First, “closed spinal dysraphism” is a category-level descriptor that encompasses many distinct entities (lipomatous and non-lipomatous, with and without cord-tethering, with and without subcutaneous extension); substituting it for “lipomyelomeningocele” would replace a precise diagnostic term with a less specific one and would compromise the manuscript’s ability to identify the particular lesion to which the described technique applies. Second, the new Orphanet codes for the specific entity described here—posterior extramedullary conus spinal cord lipoma (ORPHA:645294) and terminal extramedullary conus spinal cord lipoma (ORPHA:645288)—are now provided in the Abstract, Introduction, and this Discussion sub-section, so that concordant indexing with the SBoD framework is preserved. Third, “lipomyelomeningocele” remains the predominant term in the contemporary surgical literature, including 2024–2025 papers cited in this manuscript; abandoning it in a technical note aimed at neurosurgical trainees would impede discoverability and cross-referencing. We hope this middle position—retaining the conventional surgical term while explicitly mapping it to the new SBoD codes throughout—will be acceptable to the reviewer.

### 5.4. Skin Incision: Vertical Versus Transverse

A vertical fusiform incision over the long axis of the lipoma is our default because it best exposes the rostral pole, where the dural penetration site must be identified. A transverse incision over the inferior pole of the lipoma is an alternative that some centers prefer for very low-lying lesions, on the grounds that a transverse scar is held away from the gluteal cleft and is less likely to be soiled in the diaper period. In our experience, with the inferior pole of the incision kept at least 2–3 cm cephalad to the anal verge, wound complications have not been a particular problem.

### 5.5. Choice of Duraplasty Substrate

Autologous tissue (pericranium, fascia lata, or lumbosacral fascia) is the gold standard substrate for duraplasty in dysraphism surgery, both because of its excellent biocompatibility and because of theoretical concerns that some non-autologous materials may promote epidural fibrosis and contribute to re-tethering [11,15]. When autologous tissue is not available in sufficient quantity, an acellular biological xenograft (porcine small intestinal submucosa, e.g., Surgisis^®^) is, in our hands, a reasonable second-line option; this is a biologically derived scaffold that is gradually replaced by host tissue and is distinct from truly synthetic prosthetic dural materials, which are avoided in this context.

### 5.6. Rationale for Selective Use of a Subcutaneous Closed-Suction Drain

Reviewers 1 and 2 questioned the routine placement of a closed-suction subcutaneous drain in this procedure. We have given this point careful consideration. Resection of a large subcutaneous lipoma along a relatively long lumbosacral incision predictably creates a three-dimensional dead space deep to the skin and superficial to the reconstructed lumbosacral fascia. Several reports document that this dead space can give rise to large subcutaneous exudates or seromas; Cao et al. [20], for example, describe a case in which more than 1000 mL of subcutaneous fluid required suction drainage after lipomyelocele surgery, and such collections may compromise wound healing, generate pressure on the dural closure, and predispose to dehiscence. Our practice is therefore to place a short-term closed-suction drain under direct vision, well away from the dural closure, with the explicit goals of obliterating sub-fascial dead space and minimizing subcutaneous fluid accumulation. The drain is not in continuity with the subarachnoid space, is monitored daily, and is removed promptly once output declines (typically within 48–72 h), which limits any potential role as a conduit for retrograde wound contamination. We accept that the question of routine drainage in this population is unresolved, and we have retained the drain in our practice while acknowledging the reviewers’ valid concerns and clarifying the safeguards above.

### 5.7. Rationale for a Single-Level Laminectomy in the Lumbosacral Region

Reviewer 3 raised the concern that bilateral laminectomy in a pediatric patient may predispose to post-laminectomy kyphosis, and proposed osteoplastic laminotomy with bone-flap replacement as a preferable alternative. We agree that, for multi-level pediatric posterior spinal exposures (e.g., spinal cord tumor surgery), laminoplasty offers important biomechanical advantages and is the standard of care. The situation in lipomyelomeningocele surgery is, however, anatomically distinct in two respects. First, the posterior elements at the level of the dysraphism are already congenitally deficient (osseous schisis), so that the lipoma exits the spinal canal through a pre-existing bony defect; no additional bony work is required at the level of the lesion itself. Second, the laminectomy described here is limited to a single level immediately rostral to the defect, and its sole purpose is to identify a segment of intact, healthy dura covering normal-caliber cord through which the dural opening can be initiated safely. In our practice, this single-level minimal exposure has not been associated with clinically significant post-laminectomy spinal deformity in this patient population [11]. We have therefore retained the bilateral single-level laminectomy as described, while explicitly acknowledging the reviewer’s concern, and we have added the rationale to Step 4.

## 6. Conclusions

Pediatric LMMC surgery is technically demanding and requires a careful balance between adequate untethering/debulking of the lipoma and preservation of the placode and lumbosacral nerve roots. The 14-step approach described here—planned skin incision, identification of the dural defect, rostral-to-caudal dural opening over normal anatomy, controlled lipoma debulking with electrophysiological mapping, filum stimulation and division, pia-to-pia placode reconstruction, and expansile duraplasty with multilayered watertight closure—is consistent with contemporary published techniques and reported outcomes [3,11,14,15,21]. Long-term multidisciplinary follow-up, including periodic MRI and urodynamic surveillance, is essential, as some patients experience late deterioration despite an initially successful operation [1,9,17,22].

## Data Availability

Data cannot be shared publicly due to the violation of patient privacy and the absence of informed consent for data sharing. The corresponding author (Seung Ho Yang) had full access to all the data in the study and takes responsibility for the integrity of the data and the accuracy of the data analysis.
